# The elevation of serum napsin A in idiopathic pulmonary fibrosis, compared with KL-6, surfactant protein-A and surfactant protein-D

**DOI:** 10.1186/1471-2466-12-55

**Published:** 2012-09-11

**Authors:** Takuya Samukawa, Tsutomu Hamada, Hirofumi Uto, Masakazu Yanagi, Go Tsukuya, Tsuyoshi Nosaki, Masahiro Maeda, Takashi Hirano, Hirohito Tsubouchi, Hiromasa Inoue

**Affiliations:** 1 Department of Pulmonary Medicine, Graduate School of Medical and Dental Sciences, Kagoshima University, 8-35-1 Sakuragaoka, Kagoshima, 890-8520, Japan; 2 Department of Digestive and Lifestyle Related Disease, Graduate School of Medical and Dental Sciences, Kagoshima University, Kagoshima, Japan; 3 Department of Surgical Oncology and Digestive Surgery, Graduate School of Medical and Dental Sciences, Kagoshima University, Kagoshima, Japan; 4 Department of Research & Development, Immuno-Biological Laboratories Co., Ltd, Fujioka, Gunma, Japan; 5 Todachuo General Hospital, Toda, Saitama, Japan

**Keywords:** Biomarker, Idiopathic interstitial pneumonia, KL-6, Napsin A, SP-A, SP-D

## Abstract

**Background:**

Napsin A, an aspartic protease, is mainly expressed in alveolar type-II cells and renal proximal tubules and is a putative immunohistochemical marker for pulmonary adenocarcinomas. This study sought to determine whether napsin A could be measured in the serum to evaluate its relationship to idiopathic pulmonary fibrosis (IPF) and determine whether renal dysfunction might affect serum napsin A levels.

**Methods:**

Serum levels of napsin A were measured in 20 patients with IPF, 34 patients with lung primary adenocarcinoma, 12 patients with kidney diseases, and 20 healthy volunteers. Surfactant protein (SP)-A, SP-D, and Krebs von den Lungen-6 (KL-6) levels in serum and pulmonary function tests were also evaluated in IPF patients.

**Results:**

Circulating levels of napsin A were increased in patients with IPF, as compared with healthy controls, and they correlated with the severity of disease. Moreover, the serum napsin A levels were not elevated in patients with pulmonary adenocarcinoma or renal dysfunction. The distinguishing point between IPF and the controls was that the area under the receiver operating characteristic curve (ROC) of napsin A was larger than that of KL-6, SP-A, or SP-D.

**Conclusion:**

These findings suggest that serum napsin A may be a candidate biomarker for IPF.

## Background

Idiopathic pulmonary fibrosis (IPF), a chronic, progressive, fibrotic interstitial lung disease (ILD) with a poor prognosis, is largely unaffected by currently available medical treatments [1]. IPF is associated with the histopathologic and/or radiologic pattern of a usual interstitial pneumonia (UIP). It is characterized by progressive worsening of dyspnea and lung function. The incidence and mortality of IPF are increasing [1,2], and the median survival time is 2 to 3 years from the time of diagnosis [3]. Identification of peripheral blood biomarkers may facilitate the diagnosis, estimation of prognosis, and selection and evaluation of a treatment as well as the development of new therapeutic intervention. A number of candidate blood biomarkers for IPF including cytokines, chemokines, enzymes, collagen relevant products and products of type II epithelial cells, have been studied for their diagnostic and predictive values. Serum levels of mucin-like glycoprotein Krebs von den Lungen 6 antigen (KL-6) [4], surfactant protein (SP)-A and SP-D [5,6], matrix metalloproteinase (MMP)1 and MMP7 [7], brain natriuretic peptide [8], and, most recently CC-chemokine ligand 18 [9] are elevated in patients with IPF. KL-6, SP-A, and SP-D in blood are considered to derive from proliferating epithelial cells and/or disruption of the epithelial barrier.

Napsin A, an aspartic proteinase, is expressed in type II pneumocytes and in alveolar macrophages presumably secondary to phagocytosis [10,11]. It is abundant and active in the alveolar space, correlating with the levels of SP-B, proSP-B, and SP-C [11]. Therefore, it is possible that circulating napsin A may increase upon type II pneumocyte hyperplasia and/or epithelial barrier breakdown, such as IPF and acute lung injury. In addition, immunohistochemistry for napsin A marks most cases of lung adenocarcinomas and is negative in most squamous cell carcinomas and adenocarcinomas of other organs [12,13]. Its local expression is reported to be useful both for classifying primary lung tumors as adenocarcinoma and for identifying lung origin in the setting of a metastatic adenocarcinoma [12,13].

We hypothesized that serum napsin A levels would be increased in patients with IPF and would correlate with severity of disease [14,15]. To test this hypothesis, we quantitated levels of circulating napsin A in patients with IPF, primary pulmonary adenocarcinomas, and controls, after that we analyzed the correlations between the serum levels of napsin A and those of KL-6, SP-A, SP-D respectively, and lung function as measured by percent-predicted forced vital capacity (FVC) in IPF patients. Furthermore, napsin A is also expressed in the proximal convoluted tubules of the kidney [10], and we measured serum napsin A levels in patients with kidney disease to determine whether renal dysfunction might affect serum Napsin A levels.

## Methods

### Study subjects

We evaluated 40 ILD patients according to a flowchart, Diagnostic Process in diffuse pulmonary lung diseases (DPLD) (2002) [16]. Of these 40 patients, 10 patients were excluded due to collagen vascular disease. Of the remaining 30 patients, who were regarded as having idiopathic interstitial pneumonia (IIP), 17 patients were diagnosed with IPF based on history, physical examination, pulmonary function tests, arterial blood gas analysis, and high-resolution computed tomography of the chest. The other 13 patients underwent biopsy. Of these 13 patients, three had UIP and ten had non-UIP; of the non-UIP patients, eight had nonspecific interstitial pneumonia and two had cryptogenic organizing pneumonia. The three UIP patients were clinically consistent with a diagnosis of IPF. Consequently, 20 (17 + 3) patients met the consensus definition of IPF in accordance with Diagnostic Process in DPLD. The biopsy rates were 43% (13/30) for IIP patients and 15% (3/20) for the 20 patients in whom IPF were suspected. Serum samples were collected from 20 patients with IPF (18 males and 2 females, mean age, 72.4 ± 5.3 yr), 34 patients with primary lung adenocarcinoma without ILD (18 males and 16 females, mean age, 68.9 ± 9.4 yr), 12 patients with kidney disease (4 males and 8 females, mean age, 44.3 ± 18.2 yr), and 20 control subjects (10 males and 10 females, mean age, 60.0 ± 4.6 yr). Pulmonary function was measured by spirometry, and the mean percent-predicted FVC of IPF patients was 74.0 ± 15.7%. In 16 of 20 IPF patients, spirometry and measurement of serum napsin A were performed at the same time points. All patients in the lung cancer group underwent surgery, allowing determination on histological features and surgical TNM criteria. The patients with primary lung adenocarcinoma included thirteen with stage IA disease, three with stage IB, seven with stage IIA, seven with stage IIIA, three with stage IIIB, and one with stage IV. Preoperative serum samples were collected from patients with lung cancer. Patients with kidney disease included four with lupus nephritis, three with IgA nephropathy, two with anti-neutrophil cytoplasmic autoantibody (ANCA)-associated nephropathy, one with diabetic nephropathy, one with amyloid nephropathy, and one with interstitial nephritis in Sjogren syndrome. The mean serum creatinine level in all patients with kidney disease was 2.1 ± 1.4 mg/dl. The 20 control subjects were healthy volunteers with no evidence of comorbidity. This study was approved by the ethics committee of the Kagoshima University Graduate School of Medical and Dental Sciences (Number 21–48), and informed, written consent was obtained from patients.

### Measurements of napsin A, KL-6, SP-D, and SP-A

Serum samples collected from all groups prior to the 2011 release of guidelines, and all samples were stored at – 80°C until use. Subsequent analysis was blinded to clinical status. Levels of napsin A in sera were quantified by sandwich-type enzyme-linked immunosorbent assay, using commercially available ELISA kit (Human Napsin A Assay Kit-IBL, Japan). Serum samples with napsin A levels exceeding the top value of standard curve for the kits value were diluted and reassayed. Serum KL-6, SP-D, and SP-A were measured using commercially available ELISA kits (Eitest KL-6 kit, Sanko Junyaku, Tokyo, Japan; SP-D kit, YAMASA EIA, Yamasa, Japan; SP-A test Kokusai-F kit, International Reagents Corporation, Japan) [17-19].

### Statistical analysis

Data are expressed as means ± standard deviations. Differences in serum levels of each marker between subject groups were analyzed by ANOVA with Scheffe post hoc test or by the Student’s *t*-test. Serum levels of napsin A, KL-6, SP-A, and SP-D were further analyzed using a ROC curve to determine the appropriate cut-off level resulting in optimal diagnostic accuracy. Correlation was performed using Spearman’s rank order correlation. Significance was defined as p < 0.05.

## Results

### Serum napsin A was elevated in IPF but not in adenocarcinoma or kidney disease

No significant difference was found between in age between IPF and lung cancer patients. There were significant differences in age between control subjects and IPF patients and between control subjects and lung cancer patients, and in gender among the groups. However, no correlation was found between serum napsin A level and age, or gender in control subjects.

**Figure 1:**
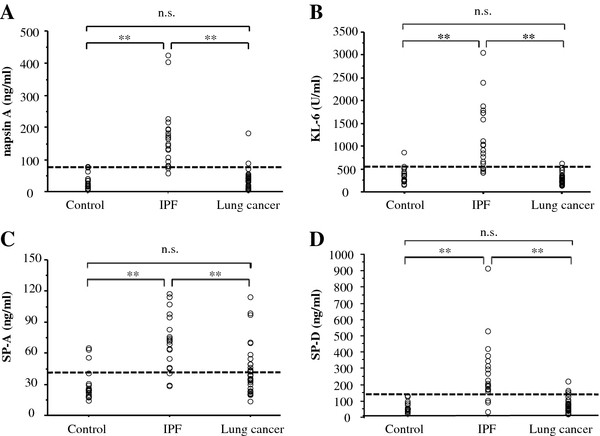
**Distribution of serum napsin A (A), KL-6 (B), SP-A (C), and SP-D (D) levels in patients with interstitial lung disease (IPF, n = 20), patients with primary pulmonary adenocarcinoma (lung cancer, n = 34), and healthy volunteers (control, n = 20).** Each horizontal line represents the diagnostic cut-off level (78.5 ng/ml for napsin A, 555.0 U/ml for KL-6, 42.8 ng/ml for SP-A, and 131.0 ng/ml for SP-D). IPF, idiopathic pulmonary fibrosis; SP-A, surfactant protein A; SP-D, surfactant protein D. **: p < 0.01. n.s.: not significant.

**Figure 2:**
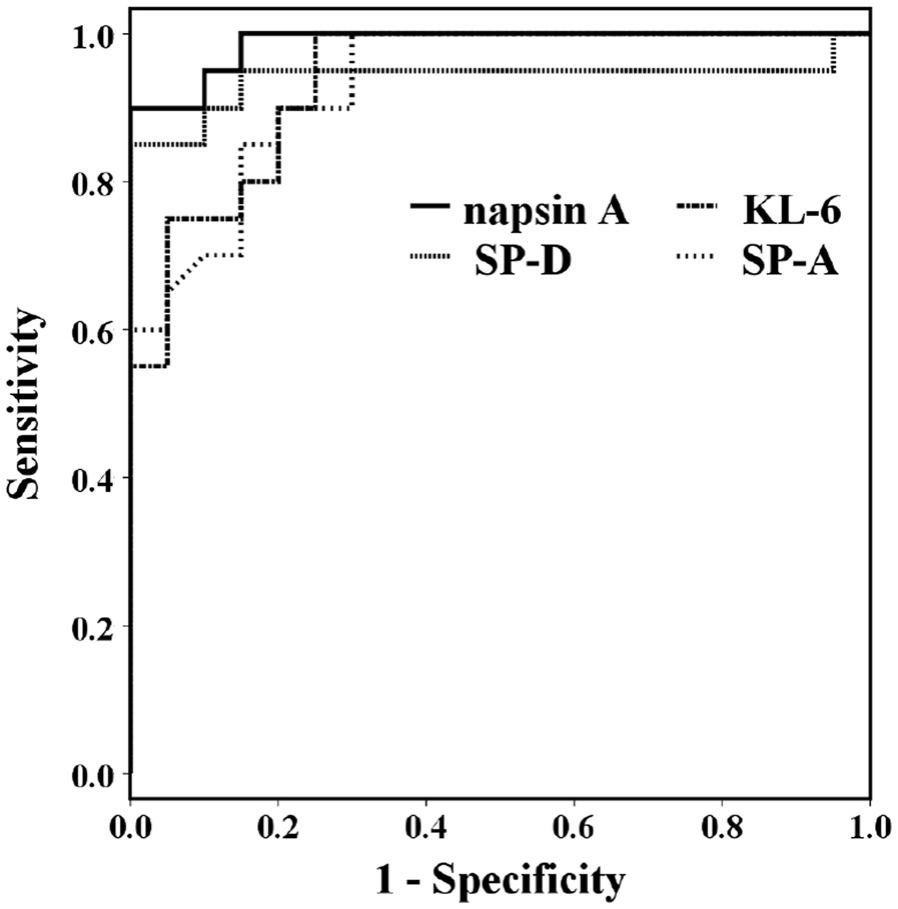
ROC curves using napsin A, KL-6, SP-A, and SP-D as serum markers for IPF in comparison with controls.

Serum Napsin A, KL-6, SP-D, and SP-A levels (Figure 1) were significantly higher in IPF patients than in control subjects and in lung cancer patients. Serum napsin A, KL-6, and SP-A levels were also significantly higher in IPF patients than in patients with lung cancer. The diagnostic values for serum napsin A, KL-6, SP-A, and SP-D for IPF vs control subjects were evaluated from the ROC curves (Figure 2). The areas under the ROC curves for IPF patients in comparison with control subjects were 0.988 for napsin A, 0.938 for KL-6, 0.931 for SP-A, and 0.940 for SP-D, with serum napsin A levels showing the greatest area, however, there were no significant differences in AUC values between serum napsin A and the other markers. The diagnostic cut-off levels using ROC curves were set at 78.5 ng/ml for Napsin A, 555.0 ml/UL for KL-6, 42.8 ng/ml for SP-A and 131.0 ng/ml for SP-D. For the diagnosis of IPF, the diagnostic accuracy of each marker determined from these cut-off levels were, 95.0% for napsin A, 85.0% for KL-6, 85.0% for SP-A, and 92.5% for SP-D. All these serum aids returned low false-positive rates in the diagnosis of IPF when compared with control subjects: 0% (0/20) for napsin A and SP-D, 5% (1/20) for KL-6, and 15% (3/20) for SP-A. SP-A showed the highest false positive rates in the diagnosis IPF vs control; false-positive rates for SP-A in lung cancer patients were unacceptably high at 35.3% (12/34). False-positive rates for other markers in patients with lung cancer were 5.8% (2/34) for napsin A, 2.9% (1/34) for KL-6 and 8.8% (3/34) for SP-D.

**Figure 3:**
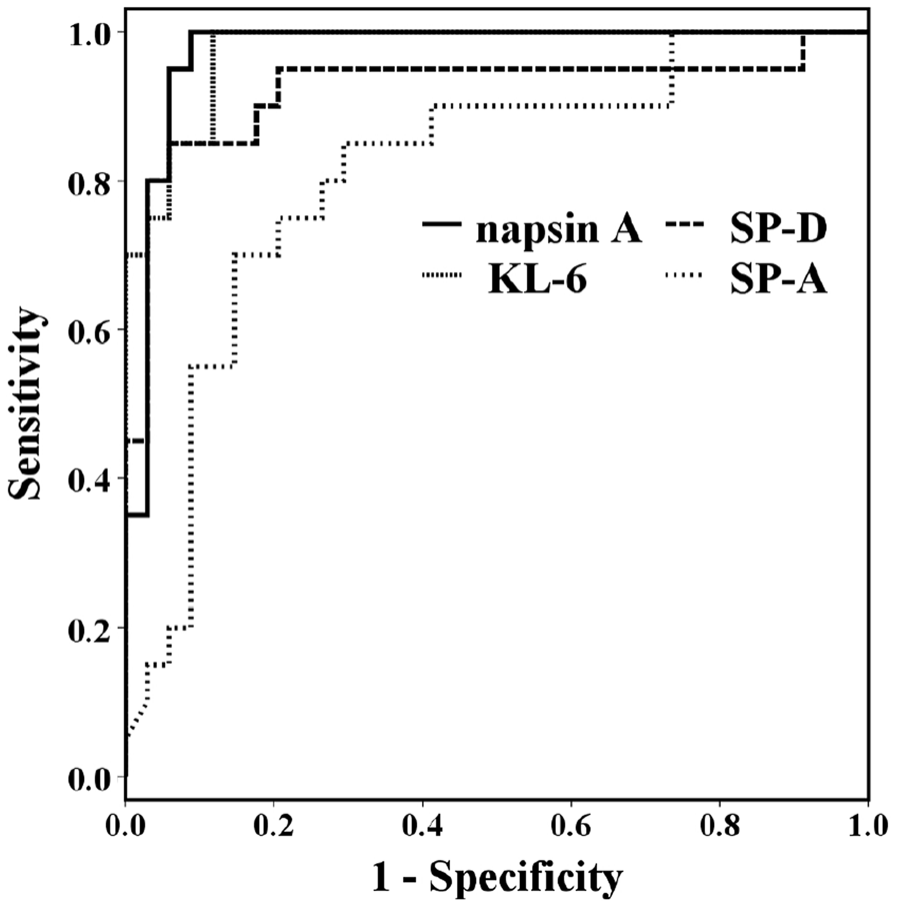
ROC curves using napsin A, KL-6, SP-A, and SP-D as serum markers for IPF in comparison with lung cancer.

**Figure 4:**
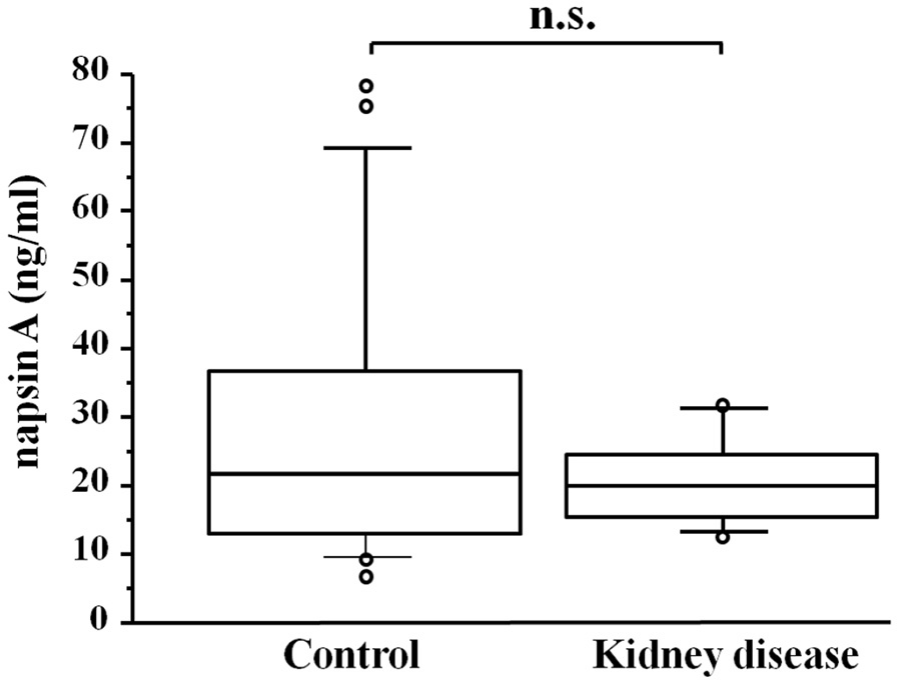
**Distribution of serum napsin A levels in 12 patients with kidney disease and 20 control subjects.** Mean level of serum creatinine of all patients with kidney disease was 2.1 ± 1.4 mg/dl. n.s.: not significant.

The diagnostic values of serum napsin A, KL-6, SP-A, and SP-D as specific markers to distinguish IPF from lung cancer were determined from the ROC curves (Figure 3). AUC values were 0.974 for napsin A, 0.975 for KL-6, 0.810 for SP-A, and 0.922 for SP-D. Napsin A and KL-6 were of greater use than SP-A and SP-D as serum markers to discriminate IPF from primary lung adenocarcinoma. As tumor markers for lung adenocarcinoma, these showed no significant difference in lung cancer vs control subjects (Figure 1). The cut-off levels for napsin A and AUC obtained from the ROC curve were 78.5 and 0.988 for IPF vs control and 76.4 and 0.974 for IPF vs lung cancer. None of the patients with kidney disease showed significant elevation of serum napsin A level in a comparison with the control subjects (Figure 4).

### Serum napsin A levels correlate with those of KL-6, SP-A, and SP-D in patients with IPF

**Figure 5:**
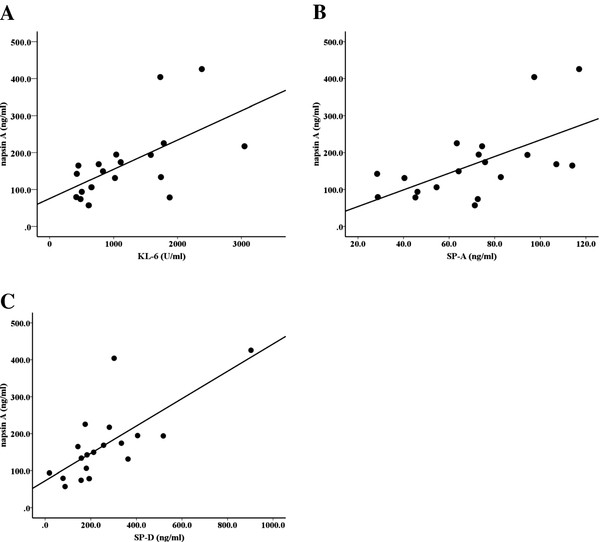
Correlation between napsin A levels and KL-6 levels (Spearman r = 0.611, P < 0.01, A), SP-A levels (Spearman r = 0.706 P < 0.01, B), and SP-D levels (Spearman r = 0.730, P < 0.01, C) in patents with IPF.

In patients with IPF, there were significant correlations between the serum napsin A levels and those of KL-6, SP-A, and SP-D (Figure 5). The correlation between napsin A levels and KL-6 levels (r = 0.611, p < 0.01), SP-A levels (r = 0.760, p < 0.01), SP-D levels (r = 0.730, p < 0.01) respectively. The serum napsin A levels in patients with IPF were more strongly correlated with SP-A and SP-D levels than with KL-6 levels.

### Napsin A levels correlate with IPF severity

**Figure 6:**
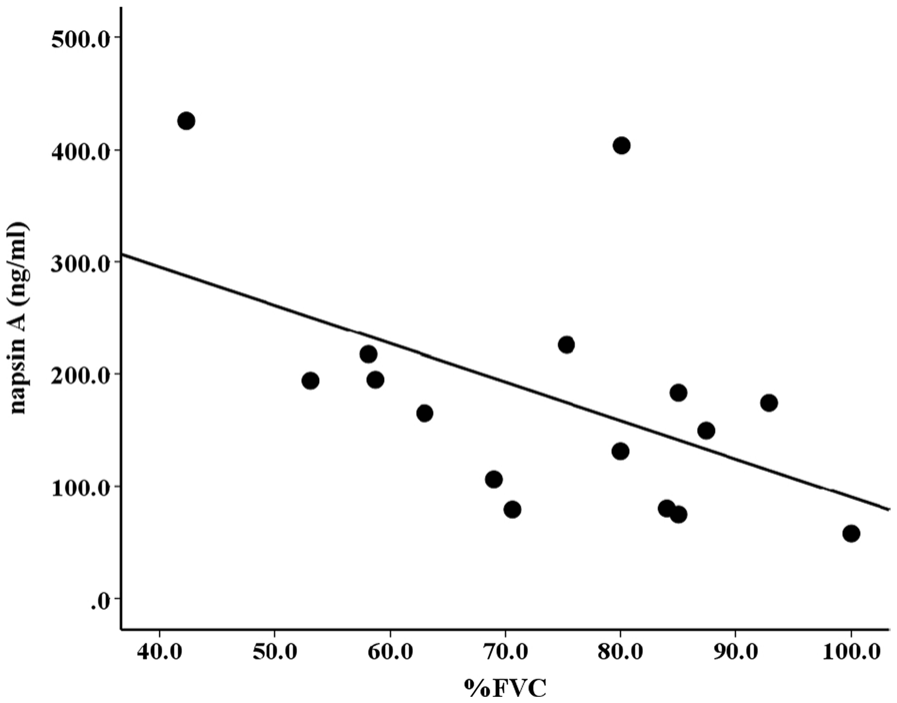
Inverse correlation between napsin levels and lung function as measured by percent-predicted FVC (% FVC) (n = 16, r = − 0.53, p < 0.05).

To determine whether napsin A levels correlate with disease severity, we compared pulmonary function measurements with serum concentrations of napsin A in IPF patients. There was moderate inverse correlation between the napsin A level and lung function as measured by percent-predicted FVC (Spearman r = −0.53, p < 0.05) (Figure 6). We did not find any statistically significant correlation between the napsin A levels and forced expiratory volume in one second (FEV_1_) values (data not shown).

## Discussion

In the present study we demonstrated that circulating levels of napsin A are increased in patients with IPF, as compared with healthy controls, and correlate with those of KL-6, SP-A, SP-D, and the severity of disease. In addition, the serum napsin A levels were not elevated in patients with pulmonary adenocarcinoma without ILD or in kidney disease. These findings suggest that serum napsin A may be a candidate biomarker for IPF.

Our findings demonstrating elevated serum levels of KL-6, SP-A, and SP-D in IPF are consistent with those reported previously [4-6], as well as the cut-off levels in this study for KL-6, SP-A and SP-D were similar to those in previous reports [17-19]. Compared to these serum markers, napsin A showed the largest AUC for distinguishing IPF from controls. A comparison of KL-6, SP-A, and SP-D for the diagnostic values in patients with ILD including IPF previously demonstrated that KL-6 was superior to other markers [6], and the findings of present study for IPF regarding the order of the AUC values obtained from ROC curves is the same as that study [6], in which KL-6 preceded SP-A and SP-D. In our findings, serum napsin A levels showed greater diagnostic accuracy for distinguishing IPF from controls.

The mechanism by which the circulating levels of napsin A are elevated in IPF is not known. It is probably due to a combination of a loss of integrity of the epithelial barrier caused by lung injury and an increased mass of type II cells due to hyperplasia as SP-A and SP-D [20]. The molecular weights of SP-A and SP-D are 26–38 kDa and 43 kDa, respectively [20-22]; that of napsin A is approximately 38 kDa [12], while that of KL-6 is estimated to be greater than 200 kD [20,23]. Serum KL-6 possibly requires cleavage by a proteinase to liberate its extracellular domain in order to leak into the bloodstream [20,24]. These differences may account for differences in the detected levels of these markers in IPF.

Serum napsin A levels were correlated with serum KL-6, SP-A, and SP-D in patients with IPF. Moreover, we found that napsin A levels were more strongly correlated with SP-A, and SP-D levels than with KL-6 levels, and this is supported by previous findings that napsin A is protease that relates to maturation of SP-B and SP-C [11]. Consequently, napsin A is also a useful type II pneumocytes marker, as it the case with existing biomarker for IPF: KL-6, SP-A, and SP-D. The serum markers for IPF are similar to those for type II pneumocytes; it is possible that these biomarkers reflect type II pneumocyte activity.

A concern regarding serum biomarkers is that elevated levels of some markers can be found in IPF as well as in malignancies, while these diseases may coincide. Serum levels of KL-6 or VEGF were reported to be increased in patients with IPF but also in lung cancer patients [25,26]. The production of SP-A and SP-D by lung adenocarcinoma cells obtained from malignant pleural effusions has also been previously reported [27]. In lung tumors, the sensitivity and specificity of napsin A immunostaining are high for identifying adenocarcinomas [12,13,28-30]. We compared the serum levels of napsin A, KL-6, SP-A, and SP-D in patients with IPF and primary pulmonary adenocarcinomas. The ROC curves demonstrated that napsin A, KL-6 and SP-D were superior to SP-A as serum markers distinguishing IPF from adenocarcinomas. The limitation in falsely positive cases with lung cancer may be able to be corrected by using in combination.

In addition to type II pneumocytes, napsin A is expressed in the epithelium of the proximal and convoluted tubules of the kidney [31]. In this study, none of the subjects with IPF, lung cancer, or controls exhibited any signs of renal dysfunction or renal cell carcinoma. Serum napsin A levels of patients with kidney disease indicated no elevation compared with those of control subjects. Therefore, it is unlikely that our data were influenced by kidney disease.

There were some limitations in this study. The study was retrospective and included only limited numbers of patients. The role of napsin A in the pathogenesis of lung disease is unknown, and it is possible that several other diseases including other types of ILD and pneumonia can cause an increase in serum napsin A levels. Therefore, a large cohort study will be required to confirm our results. We will also need to clarify the relationship of these markers to the histological patterns of ILD.

## Conclusions

We have shown that napsin A is found in increased quantities in the circulation of patients with IPF, in whom the levels correlate with those of KL-6, SP-A, SP-D, and lung function. Napsin A is superior to KL-6, SP-A and SP-D for distinguishing IPF from controls. Although these findings do not allow us to determine whether napsin A is useful for predicting the outcome in IPF yet, they support the hypothesis that napsin A is a candidate biomarker for diagnosing the presence of disease in an individual.

## Abbreviations

IPF: Idiopathic pulmonary fibrosis; SP-A: Surfactant protein A; SP-D: Surfactant protein D; ROC: Receiver operating characteristic curve; F**VC**: Forced vital capacity; FEV_1_: Forced expiratory volume in 1 second.

## Competing interests

All authors except for Masahiro Maeda have no potential conflicts of interest exist with any companies/organizations. Masahiro Maeda is an employee of Immuno-Biological Laboratories.

## Authors’ contributions

TS: contributed to the planning, data collection, data analysis, and writing of the manuscript. TH: contributed to data collection, data analysis, and writing of the manuscript. HU: contributed to data analysis, and writing of the manuscript. MY: contributed to data collection, data analysis, and writing of the manuscript. GT: contributed to data collection, data analysis, and writing of the manuscript. TN: contributed to data collection, data analysis, and writing of the manuscript. MM: contributed to data analysis, and writing of the manuscript. TH: contributed to data analysis, and writing of the manuscript. HT: contributed to data analysis, and writing of the manuscript. HI: contributed to the planning, data collection, data analysis, and writing of the manuscript. All authors read and approved the final manuscript.

## Pre-publication history

The pre-publication history for this paper can be accessed here:

http://www.biomedcentral.com/1471-2466/12/55/prepub
